# Thermoreversible Diels–Alder Cross-Linking of BHMF-Based Polyesters: Synthesis, Characterization and Rheology

**DOI:** 10.1021/acssuschemeng.4c09338

**Published:** 2025-02-27

**Authors:** Cornelis Post, Paul van den Tempel, Paula Herrera Sánchez, Dina Maniar, Ranjita K. Bose, Vincent S. D. Voet, Rudy Folkersma, Francesco Picchioni, Katja Loos

**Affiliations:** † University of Groningen, Zernike Institute for Advanced Materials, Macromolecular Chemistry & New Polymeric Materials, Nijenborgh 3, AG Groningen 9747, The Netherlands; ‡ Circular Plastics, Academy Tech & Design, NHL Stenden University of Applied Sciences, Van Schaikweg 94, KL Emmen 7811, The Netherlands; § Department of Chemical Engineering, University of Groningen, Engineering and Technology Institute Groningen (ENTEG), Nijenborgh 3, AG Groningen 9747, The Netherlands

**Keywords:** 2,5-bis(hydroxymethyl)furan, Diels–Alder, bismaleimide-689, biobased polymers, enzymatic polymerization, rheology

## Abstract

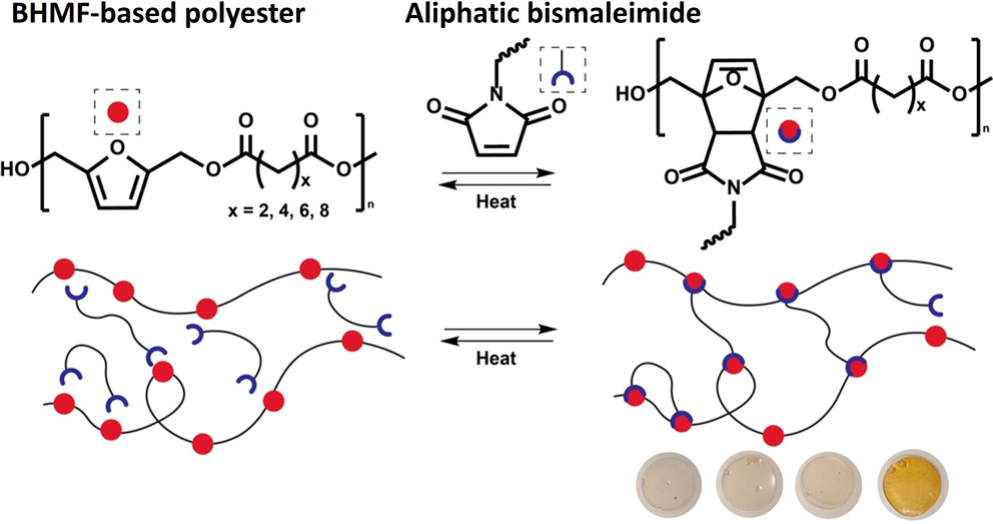

2,5-Bis(hydroxymethyl)furan (BHMF)-based polyesters are an interesting new class of biobased and potentially biodegradable furanic polymers. However, their thermal properties are currently insufficient, making them unsuitable for potential applications as commodity plastics. To improve this, several polyesters were synthesized using an enzymatic bulk polymerization process with the monomers BHMF and aliphatic dimethyl esters of varying spacer lengths. The BHMF units were subsequently cross-linked with a biobased bismaleimide (BM-689) through a [4 + 2] cycloaddition reaction between the furan and maleimide groups. This approach clearly demonstrated that these furan-based polyesters can be thermoreversibly cross-linked using this bismaleimide. The use of different spacer lengths further allows the variation in relative reaction rates and affects the reversibility and reprocessability of the polymers. In situ ^1^H NMR spectroscopy revealed the identification of the endo- and exostereoadducts formed by the Diels–Alder reaction. A rheological analysis demonstrated the reprocessability and the thermal properties were further characterized via thermogravimetric analysis (TGA) and differential scanning calorimetry (DSC). This study sheds light on the thermal and mechanical enhancement of biobased BHMF-based polyesters, while maintaining their recyclability, which widens the range of potential application of this promising polymer.

## Abstract 2

Thermoreversible cross-linking of BHMF-based polyesters with an aliphatic bismaleimide via Diels–Alder chemistry to improve thermal and mechanical properties and maintain recyclability.

## Introduction

Polymers play an essential role in daily life and are present in numerous applications in different fields.^1^ However, the majority of these plastics are produced from nonrenewable fossil resources and lead to significant greenhouse gas emissions during the production and end-of-life stages, such as incineration and recycling.^2^ Biobased polymers are considered more sustainable alternatives, due to reduced carbon dioxide emissions while maintaining the beneficial properties of plastics in our society.^3,4^ Hence, both the amount of research and the production of biobased polymers are increasing exponentially and are estimated to be 6.3 million tons by 2027 but still constitute a fraction of the total amount of plastic (400 million tons, 2022).^5,6^

A relatively new type of biobased polymers are 2,5-bis(hydroxymethyl)furan (BHMF)-based polyesters, which can be synthesized using BHMF with several different comonomers, including aliphatic diesters, lactides and other furan derivatives.^7^ BHMF is a biobased furanic compound similar to the well-known 2,5-furandicarboxylic acid (FDCA) and can be obtained from 5-hydroxymethylfurfural (HMF) or fructose as starting material in high yields.^8^ BHMF-based polyesters have been shown to feature a wide range of properties by varying the number of methylene units in the repeating unit.^9,10^ In addition, they can be produced via (enzymatic) bulk polymerization and have demonstrated biodegradable behavior in activated sludge.^11^ However, their thermal and mechanical properties are limited because of their relatively low melting points (63–98 °C) and molecular weights.

One way to improve this is through the introduction of covalent cross-linking of the chains, but this usually occurs at the expense of recyclability.^12^ A solution to this problem is the implementation of covalent adaptable networks (CANs), which are thermosets containing dynamic covalent bonds. By endowing the network with dynamic covalent bonds, it can be rearranged to temporarily break cross-links to make recycling possible.^13^ CANs can be divided into two categories: dissociative and associative dynamic bonds. In the case of associative dynamic bonds, the cross-link density remains constant as bond formation and dissociation occur simultaneously. This is not the case for dissociative dynamic bonds, where even the complete network can disintegrate into the original building blocks.^13,14^ An example of such a dissociative reaction is the [4 + 2] cycloaddition (Diels–Alder) reaction between furan and a maleimide.^15^ The reaction has become a very popular topic over the last 25 years, since the first example of a Diels–Alder-based CAN in 2002 by Wudl et al.^16^ In recent years, the Diels–Alder reaction has also been used for reversible polymer cross-linking, for example, with polyketones,^17^ lignin/cellulose networks,^18^ and rubbers.^19^ However, the Diels–Alder reaction between BHMF and a maleimide group is challenging since the aromaticity has to be broken, although it was shown to be feasible with different maleimides.^20^ In addition, the Diels–Alder reaction between FDCA units and maleimide groups is particularly challenging due to the electron-withdrawing carbonyl groups adjacent to the furan ring; however, it has been shown to be feasible for FDCA-based polyimides.^21^

Cross-linking of BHMF-based polymers via Diels–Alder chemistry therefore also seems to be an interesting opportunity to reinforce BHMF-based polymers while maintaining their recyclability. This approach was successful by Yoshie et al., who produced and cross-linked poly(2,5-furandimethylene succinate) by using five different bismaleimides with different furan maleimide ratios to study their thermal, mechanical, and self-healing abilities and shape memory behavior.^22−24^ They extended their work by using the copolymers poly(2,5-furandimethylene succinate-*co*-propylene succinate), wherein the furan content and furan to maleimide ratios were varied to increase the range of thermal and mechanical properties of these networks.^25,26^ Similar chemistry was shown by Cai et al. (2018), who synthesized copolyesters from BHMF, succinic acid and lactide to yield poly(2,5-furandimethylene succinate)-*b*-poly(l-lactide).^27^ The furan content and furan-to-maleimide ratios were varied to study the thermal properties, self-healing efficiency and mechanical properties.

Although the thermoreversible cross-linking of BHMF-based polyesters has been studied previously, it is still limited to poly(2,5-furandimethylene succinate) and furanic-aliphatic copolyesters. In addition, a detailed understanding of the reaction kinetics, recyclability, and rheological behavior is still lacking. The Diels–Alder reaction between a furan and maleimide produces two stereoadducts, the kinetically favored endoadduct and the more thermally stable exoadduct.^28,29^ The identification of these two isomers for BHMF-based polyesters also remains relatively unexplored.

**Figure 1:**
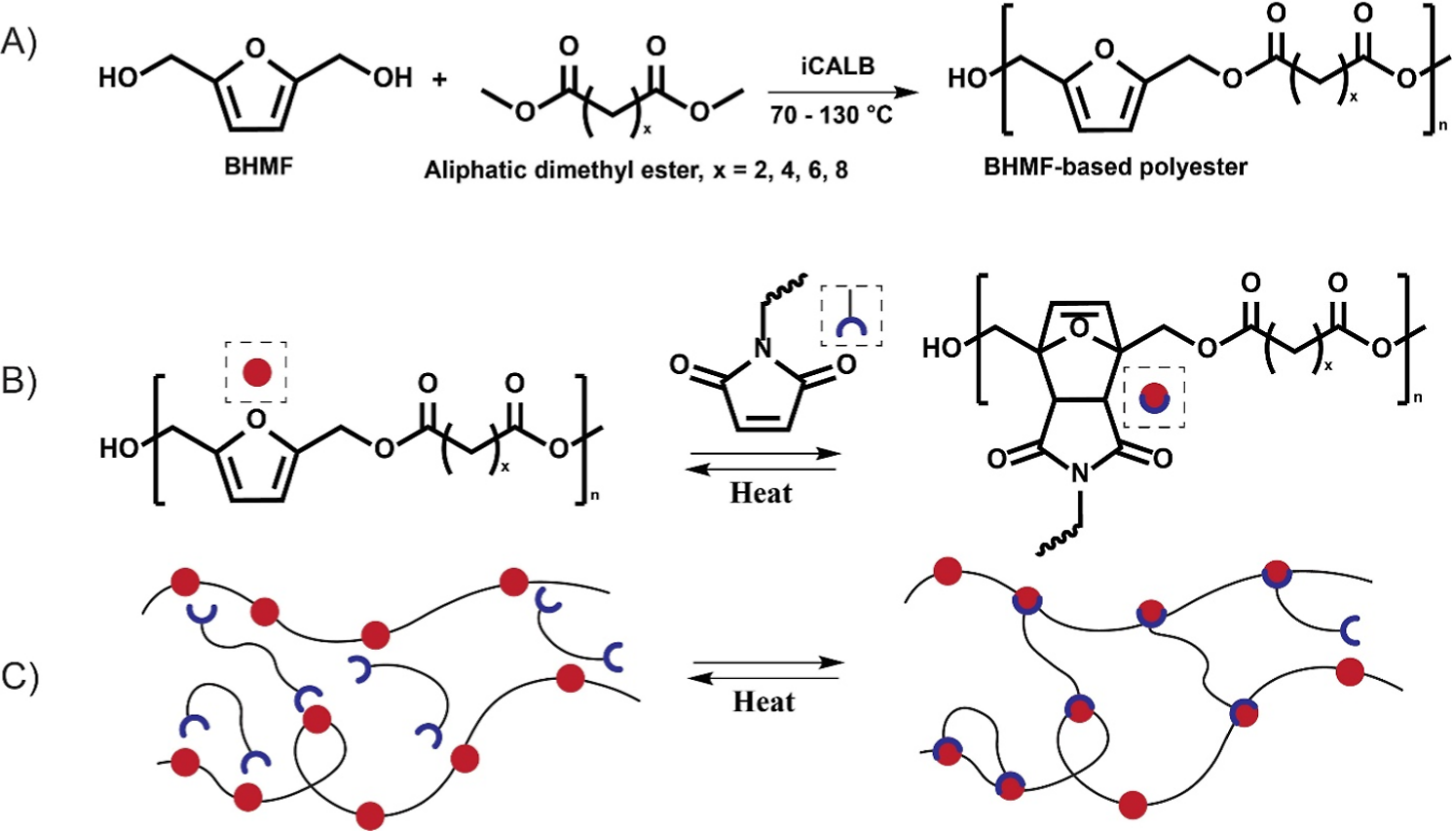
(A) Enzymatic polymerization of BHMF and aliphatic dimethyl esters of various lengths, (B) reaction of BHMF-based polyester with a maleimide group, (C) schematic overview of the thermoreversible Diels–Alder cross-linking of the furan units of the polyester with bismaleimide units.

In this work, BHMF-based polymers are enzymatically synthesized via a bulk polymerization process (Figure 1A). Enzymatic polymerizations have proven to be efficient and sustainable methods for producing a variety of biobased furan-based polyesters, polyamides and polyesteramides.^10,30−36^ In addition, the immobilized enzyme catalyst (iCALB) can be separated and recycled, which enhances the sustainability, although the activity and yield is lower and the extent of the decrease is expected to be dependent on the exposed conditions.^37^

The length of the aliphatic segment was varied to obtain structurally different polyesters with varying polymeric properties and facile control of the furan content. These polyesters are subsequently reversibly cross-linked via Diels–Alder chemistry via an aliphatic biobased bismaleimide (BM-689), which is derived from the [4 + 2] cycloaddition of fatty acids (Figure 1B,C).^38^ The effects of the number of methylene units in the aliphatic part of the polyester, i.e., the furan content, on the reversibility and reaction kinetics are studied in detail, with a particular focus on identifying the endo- and exoadducts through ^1^H NMR spectroscopy. The cross-linked polymers were analyzed by ^1^H NMR, DSC, FTIR and rheometry. The aim is to improve the mechanical properties and thermal application range of BHMF-based polyesters by introducing cross-links, while maintaining their recyclability. In addition, the focus is on illuminating the cross-linking of BHMF-based polymers, exploring the tunability of their final network characteristics, and investigating the relatively unexplored reaction kinetics of the Diels–Alder reaction within these systems.

## Experimental Section

### Materials

Dimethyl succinate (DMSuc, 98%), dimethyl adipate (DMAd, >99%), dimethyl suberate (DMSub, 99%), lipase acrylic resin [Candida Antarctica lipase B (iCALB), 5000 U/g, recombinant, expressed in *Aspergillus niger*], and chloroform (amylene stabilized, HPLC grade, >99.8%) were purchased from Sigma-Aldrich. 2,5-Bis(hydroxymethyl)furan (BHMF, >97%) was obtained from Apollo Scientific. Dimethyl sebacate (DMSeb, >98%) was purchased from TCI EUROPE, and diethyl ether was obtained from Honeywell Research Chemicals. The aliphatic bismaleimide (BM-689) was purchased from Caplinq Europe (Assendelft). All chemicals were used without any treatment.

### Polyester Synthesis

The synthesis route of the four different linear polyesters from BHMF and the aliphatic comonomers, poly(2,5-furandimethylene succinate) (PFSuc), poly(2,5-furandimethylene adipate) (PFAd), poly(2,5-furandimethylene suberate) (PFSub) and poly(2,5-furandimethylene sebacate) (PFSeb), was based on our previous work but was further optimized.^11^ Briefly, a 25 mL three-necked round-bottom flask was equipped with magnetic stirring egg and connected via a distillation setup to a Schlenk line to allow switching between a mild argon flow or high vacuum (0.02 mbar). BHMF (2.00 g, 1 equiv) was reacted with either DMSuc (2.31 g, 1.01 equiv), DMAd (2.74 g, 1.01 equiv), DMSub (3.18 g, 1.01 equiv) or DMSeb (3.64, 1.01 equiv) using 10 w/w % iCALB as a catalyst. Over the first 24 h, the flask was left at 70 °C under atmospheric pressure with an argon flow stirring at 300 rpm to prevent BHMF degradation. After this oligomerization step, the system was gently switched to vacuum and maintained for another 6 h at 70 °C, while the stirring speed was slowly reduced to 40 rpm due to the increased viscosity. The reaction temperature was subsequently increased to 100 °C, to enhance the reaction kinetics and to achieve an improved mixing behavior, maintained for 17 h and then increased to 130 °C for the remaining 7 h of synthesis. The polymerization of BHMF and DMSuc was prolonged for another 18 h to compensate for the lower reactivity, as described in our previous work.^11^ The obtained polymers were dissolved in chloroform, separated from the enzyme beads via a needle and syringe, precipitated in diethyl ether, centrifuged and allowed to dry in a fume hood. The polymers were obtained as whitish powders in good yields, ranging from 75 to 81%, and were characterized by ^1^H NMR, GPC, TGA and DSC.

### Cross-Linking with BM-689

The obtained linear polyesters were cross-linked with BM-689 via solvent casting using chloroform as the solvent. The polyester (0.50 g) and BM-689 were separately dissolved in 4 and 1 mL of chloroform, respectively, prior to mixing. The combined solutions were placed in an open mold on a preheated hot plate (55–60 °C) to allow cross-linking and evaporation of the solvent for 16 h. A final curing step was performed by placing the mold in an oven at atmospheric pressure and a similar temperature (60 °C) for another 6 h. Two different series were prepared, one with a furan to maleimide molar ratio of 1.0:0.50 and one with a molar ratio of 1.0:0.25. The cross-linked polymers are named according to the name of the polyester and the ratio of furan to maleimide, for example, PFSuc_0.5. The cross-linked films were obtained as colorless to slightly yellowish transparent films with a thickness of ≈1 mm. These materials were used for the thermal characterization, rheological analysis and the swelling tests.

### Kinetic Study

The reaction kinetics of the cross-linking of the BHMF-based polyesters and BM-689 were followed via ^1^H NMR spectroscopy. The polymer (0.0510 mmol) and BM-689 (0.0127 mmol), with a furan to maleimide molar ratio of 1.0:0.50, were separately dissolved in DMSO-*d*_6_, combined and thereafter transferred to an NMR tube. The tube was placed in a preheated 500 MHz Varian VXR spectrometer at 60 °C, and every 15 min, a measurement was taken for 16 h. The spectra were based on 32 scans and the acquisition and relaxation time were set at 2.73 and 2.00 s, respectively. The obtained spectra were processed and analyzed via the software MestReNova. The conversion was calculated using the formulas below
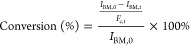
1
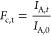
2

The integrals of the alkenyl hydrogens of BM-689 (6.96 ppm) at time 0 (*I*_BM,0_) and time *t* (*I*_BM,*t*_) where obtained from the normalized ^1^H NMR spectra and used to calculate the conversion (%). A correction factor (*F*_c,*t*_) was added to compensate for the minor decrease of the integral values, the aliphatic peak ranging from 1.0 to 1.4 ppm was used a reference, at time t (*I*_A,*t*_) and time 0 (*I*_A,0_) respectively. HeteroNuclear single quantum coherence (HSQC) and correlated spectroscopy (COSY) were performed at the end of the kinetic study to identify all signals.

### Characterization

Proton nuclear magnetic resonance (1H NMR) analyses were performed on a Varian VXR 400 MHz spectrometer, and the linear polyesters were dissolved in DMSO-*d*_6_.

The number-average molecular weight () and weight-average molecular weight () of the noncrosslinked BHMF-based polyesters were determined via gel permeation chromatography (GPC). This analysis was performed with a Malvern Viscotek GPCmax instrument equipped with a Schambeck RI2912 refractive index detector and a Malvern Dual detector. A conventional calibration curve, which is based on refractive index signals, was used to calculate the molecular weights, which were based on narrow dispersity polystyrene standards ( ranging from 625 to 3,001,000 g/mol). Two PL gel 5 mm MIXED-C 300 mm columns (Agilent Technologies) were used for separation at an operating temperature of 35 °C. Chloroform (amylene-stabilized HPLC grade) was used as the solvent and eluent at a flow rate of 0.5 mL/min. The software Viscotek OmniSec (version 5.0.) was used to operate the system and process the data. The samples were prepared at a 2 mg/mL concentration and filtered through a 0.20 μm PTFE filter prior to measurement.

Thermogravimetric analysis (TGA) was performed to evaluate the thermal stability of the linear polyesters and the cross-linked materials. A TA-Instruments Discovery TGA 5500 was used, and the samples were subjected to a heating ramp from room temperature to 700 °C at a constant rate of 10 °C/min under an inert atmosphere (N_2_).

The thermal properties of both the linear polyesters and the cross-linked materials were analyzed via differential scanning calorimetry (DSC), which was conducted on a TA-Instruments Q1000 DSC. The analysis of the linear polyesters consisted of two heating ramps and one cooling ramp of both 10 °C/min under a nitrogen environment, a temperature range from −70 to 190 °C and a sample size of 5 mg. The cross-linked samples (5 mg) were subjected to four heating and three cooling ramps, from −70 to 180 °C, under a mild nitrogen flow and a heating and cooling rate of only 2 °C/min, similar to the rheology temperature sweep experiments, as mentioned below.

The prepared cross-linked films were analyzed via attenuated total reflectance Fourier transform infrared spectroscopy (ATR-FTIR). The spectra were recorded on a Shimadzu IR-tracer-100 (Kyoto, Japan) using 32 scans and a resolution of 4 cm^–1^. The equipment contained a golden gate diamond ATR sampler equipped with a temperature control (Specac) accessory. After 3 min preheating of the sample, a spectrum was recorded on the preheated stage at 40 °C for each sample. In a separate experiment, the accessory was heated up to approximately 120 °C. Here, a spectrum was recorded 5 min after sample deposition, using 32 scans and a resolution of 4 cm^–1^. For better comparison, the spectra recorded at 120 °C were vertically corrected such that the signals in the range of 1300–1500 cm^–1^ overlap with the same signals recorded at 40 °C.

Solubility tests of the prepared samples via solvent casting were performed to evidence the network formation of the materials. The samples (15 mg, *W*_i_) were immersed in chloroform (15 mL) in triplicate for 5 days at room temperature to reach equilibrium. Subsequently, each sample was removed and the surface was dried and the swollen mass (*W*_s_) was determined. Finally, the samples were dried in the vacuum oven at 30 °C for 24 h to remove the absorbed solvent and weighed again (*W*_d_). The swelling ratio and gel fraction were determined according to formulas below.^39^
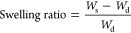
3
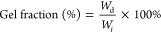
4

Rheometry experiments were conducted on a Discovery HR20 rheometer (TA-Instruments). The 25 mm discs were punched out of the prepared cross-linked films, and the experiments were carried out on a 25 mm stainless steel plate–plate geometry. The linear viscoelastic region was determined via a strain sweep at 40 °C and ranged from 0.01 to 100% at a frequency of 1 Hz. The materials were then subjected to a frequency sweep, ranging from 0.1 to 100 rad s^–1^, at 40 °C and a strain of 0.3%. Finally, the material was heated with a rate of 2 °C min^–1^ with a strain of 0.3% and a constant frequency of 1 Hz. The samples were measured at a constant gap and loaded under an axial force of 10 N. The gap was kept constant during the heating ramp.

## Results and Discussion

### Polyester Synthesis and Cross-Linking

Linear BHMF-based polyesters were synthesized via an enzymatic bulk polymerization process (Figure 1A). The procedure was based on our previous work but was optimized to reduce the reaction time while maintaining a similar molecular weight.^11^ The structure of the polymers was confirmed by GPC and ^1^H NMR analysis (Supporting Information Figures S4–S7). The number-average molecular weight  and weight-average molecular weight  range from 6400 to 10,500 and 12,800 to 17,000 g/mol, respectively (Table 1). However, the degree of polymerization (DP), ranging from 30 to 39, is more relevant since it represents the average number of furan units per chain. The value was aimed at the same order of magnitude for all four polyesters to allow for a fair comparison among the polymers during and after cross-linking with bismaleimide-689. Hence, the polymerization time of PFSuc was increased to compensate for the lower reaction kinetics, which was also observed in our previous work.^11^

**Table 1:**
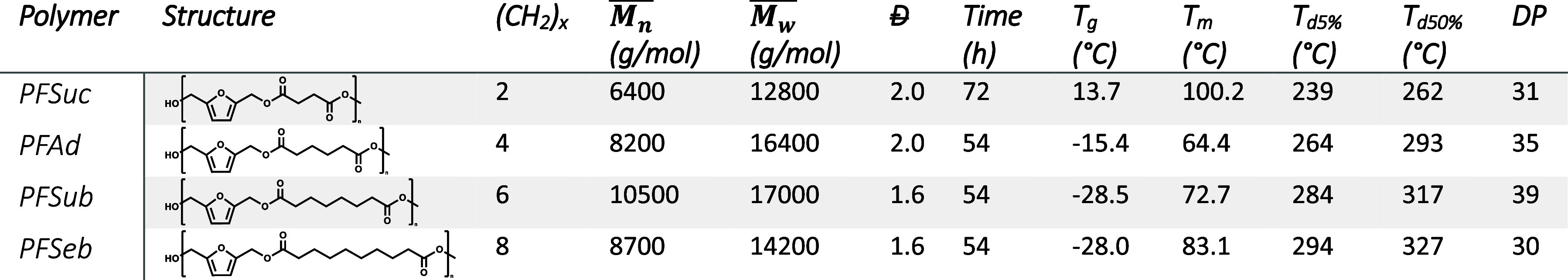
Molecular Weights, Reaction Times and Thermal Characteristics of Enzymatically Produced BHMF-Based Polyesters Containing Linear Aliphatic Segments with Different Numbers of Methylene Units

**Figure 2:**
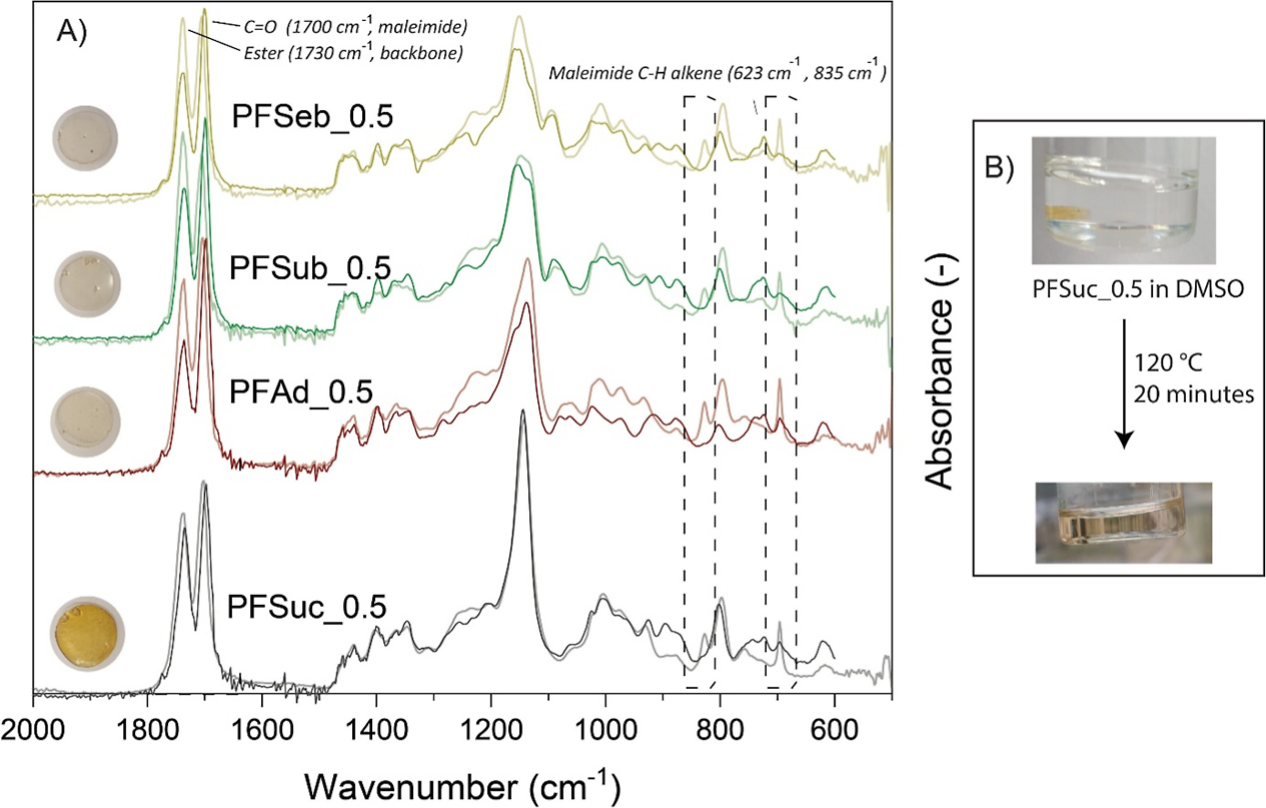
(A) Infrared spectra of four cross-linked films: PFSuc_0.5, PFAd_0.5, PFSub_0.5 and PFSeb_0.5. The solid lines display the spectra measured at 40 °C. The dashed lines are the measured spectra at 120 °C after 5 min. Photos of the cross-linked samples are included next to each spectrum. (B) Demonstration of PSuc_0.5 reversibility, 15 mg of the material was added to 15 mL DMSO showing insolubility after 5 days. Leaving the sample at 120 °C for 20 min resulted in dissociation of the network caused by the retro-Diels–Alder reaction.

The BHMF-based polyesters were cross-linked with biobased aliphatic bismaleimide-689. The cross-linked materials were prepared via solvent casting at 60 °C using chloroform as the solvent to both avoid crystallization of the polymer and achieve proper mixing of the reactants. For most of the samples, this resulted in homogeneous and transparent films (Figure 2). As opposed to mixing the polymer and bismaleimide with solvent, blending them at temperatures above the melting point of the polymer, for example via extrusion, followed by a defined cooling step is a more sustainable and green alternative to produce cross-linked materials. However, the required conditions differ for each system, which may influence the Diels–Alder reaction and prevent fair comparisons between the samples.

### Structure Confirmation and Reaction Kinetics

As displayed in Figure 2A, the FTIR spectra revealed that the double bonds of bismaleimide cannot be observed in the prepared films, as evidenced by the absence of a sharp signal at a wavenumber of 693 cm^–1^ (maleimide ring deformation).^40^ The strong band at 1730 cm^–1^ is attributed to the C=O asymmetric stretching vibration of the ester group in the polymer backbone. C=O stretching of the maleimide ring can be observed at 1700 cm^–1^. The signals between 1300 and 1500 cm^–1^ correspond to the deformation and wagging vibrations of the CH_2_ groups and the CN stretching vibrations from the maleimide.^9,19^ In this part of the spectrum, an overlap between the spectra is observed after heating the sample, which is expected from these chemical fragments that are not affected by the retro Diels–Alder reaction. After heating to 120 °C, the distinct small maleimide double bond signal can be observed again at 693 cm^–1^. This indicates the reversibility of the Diels–Alder reaction between furan units and bismaleimide, which is observed for all the prepared materials. It is known for BM-689 and other flexible multifunctional maleimides that the Diels–Alder does not completely revert back to zero conversion.^41^ The Diels–Alder bonds only revert partially instead, and the regeneration of maleimide moieties is further hindered by the bulk conditions and the excess furan units present in the system. The network formation and the reversibility of the system is also visually illustrated in Figure 2B. The material PFSuc_0.5 remained insoluble in DMSO for 5 days at room temperature. However, once heated to 120 °C, the retro Diels–Alder reactions take place and the network dissociates and the polymer and BM-689 dissolved.

**Figure 3:**
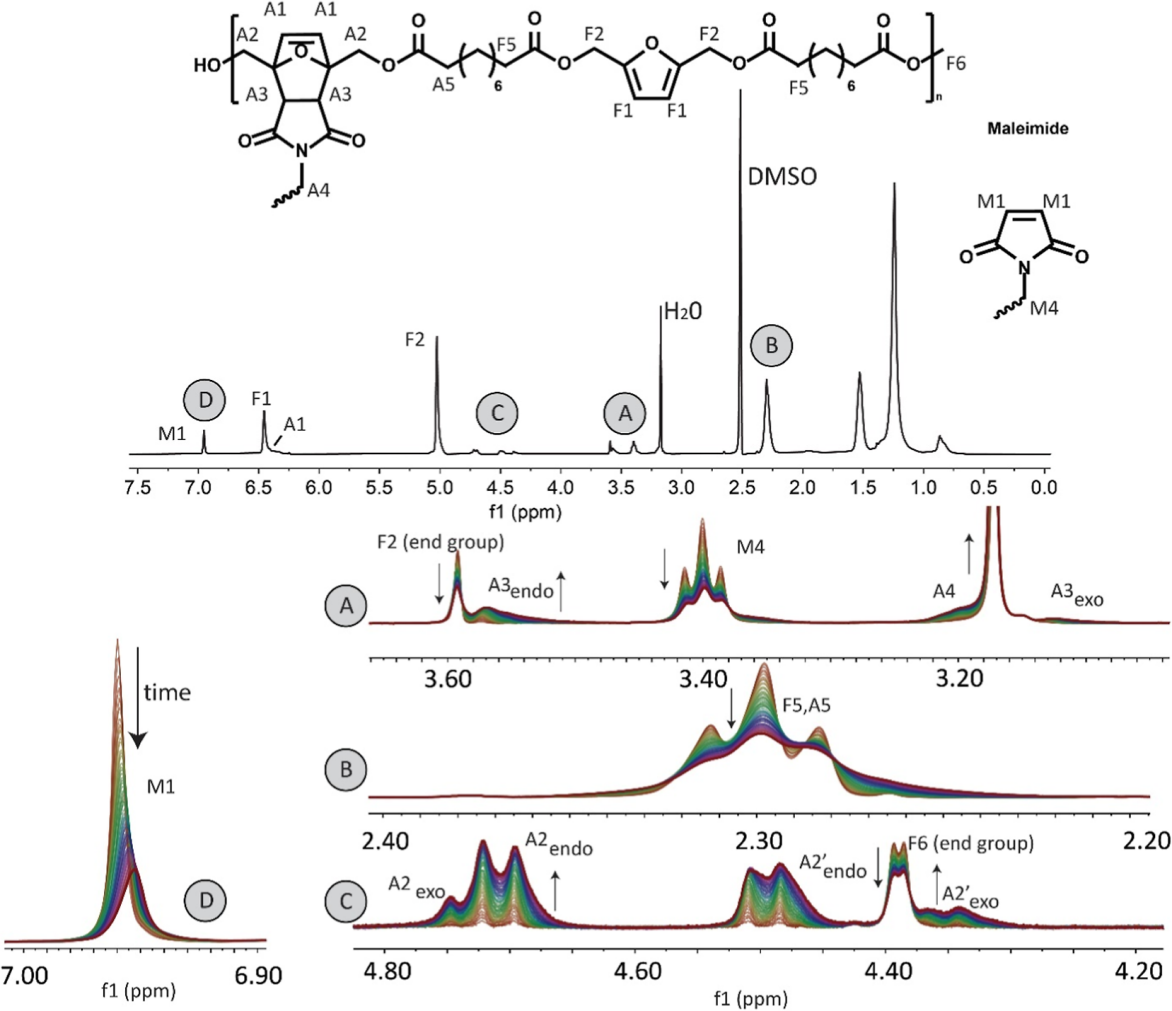
^1^H NMR results of the cross-linking process of PFSeb_0.5 (*x* = 8, furan/maleimide molar ratio is 1.0:0.5) after 16 h at 60 °C. Several regions are indicated with A, B, C, and D. These regions in the spectrum are displayed in detail and show the developments of these peaks over time.

The cross-linking process of the four synthesized polymers was investigated in solution using in situ ^1^H NMR spectroscopy. This analysis revealed the relative cross-linking rates of the structurally distinct polymers. So far, ^1^H NMR spectroscopy has proven to be the simplest method for identifying and tracking the formation of stereoadducts, specifically the endo and exoadducts.^29,42^Figure 3 shows an example of a partially cross-linked PFSeb_0.5 polymer, with the development of several signals. The consumption of bismaleimide can be observed at δ = 7.0 ppm. Interestingly, the formation of endo- and exoadducts can also be distinguished in these systems through the formation of several characteristic peaks in the region of δ = 4.2–4.8 ppm. As indicated in Figure 3, the diastereotopic protons located at position “A2” split into two signals due to the formation of a chiral carbon next to them, and the difference in chemical shift between the two signals is greater for the exoadduct than for the endoadduct. The same can be concluded from the COSY and HSQC results, where these signals show the same connectivity to the same carbon but also a COSY correlation. These spectra are included in the Supporting Information (Figures S2 and S3). The signals attributed to the endoadduct are stronger than the exoadduct signals, implying that the formation of the endoadduct dominates over the formation of exoadducts at 60 °C in DMSO. This is expected from furan-maleimide systems on the basis of previous kinetic studies.^41^ For these systems, the endoadduct is often kinetically favored over the more thermodynamically stable exoadduct. The ^1^H NMR spectra of all four polymers with BM-689 after 16 h at 60 °C in DMSO are shown in the Supporting Information (Figures S8–S11).

The kinetic study consisted of a diluted polymer bismaleimide mixture in DMSO-*d*_6_ at 60 °C, which was subjected to simple ^1^H NMR analysis every 15 min for a period of 16 h. The progress of the Diels–Alder reaction was monitored by tracking the decreasing intensity of the alkenyl protons in the maleimide groups of the unreacted BM-689 (“M4” in Figure 3, 6.96 ppm). All obtained spectra were normalized on the basis of the solvent peaks and corrected for the minor decreasing intensity of the signals, which was based on the large peak of the aliphatic groups, ranging from 1.4 to 1.0 ppm. This phenomenon was attributed to minor solubility issues of the polymers and BM-689 in DMSO or some precipitation of the reaction products. The decrease in intensity ranged from a maximum of 8–17% after 16 h.

**Figure 4:**
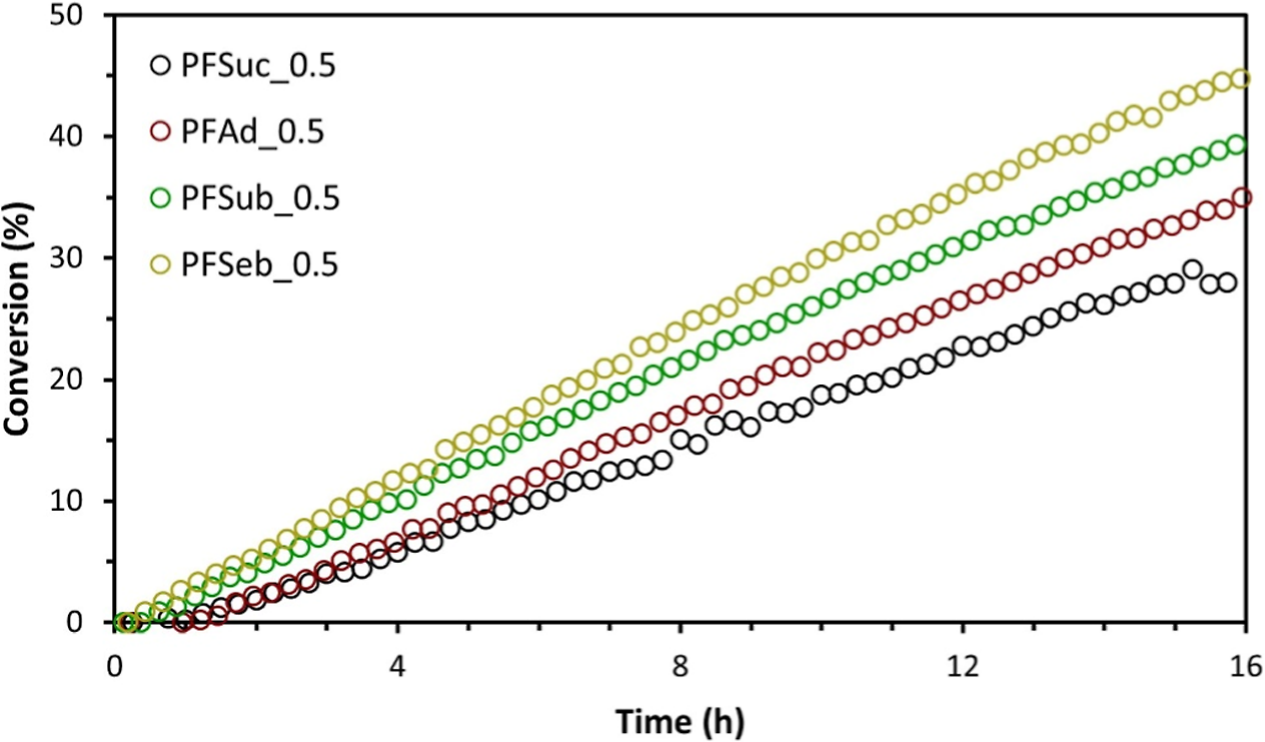
Conversion (%) of maleimide groups versus reaction time of four different BHMF-based polyesters reacting with BM-689 in DMSO-*d*_6_, as determined via ^1^H NMR analysis.

The resulting conversion of the Diels–Alder reaction versus reaction time is illustrated in Figure 4. The major observation is the clear influence of the length of the aliphatic segment of the polyester on the reaction rate. A greater number of CH_2_ groups in the repeating units resulted in a significantly increased conversion of maleimide groups after 16 h. This could be explained by the accessibility of the furan units. The effect of the aliphatic spacer as a side group normally counteracts the fast occurrence of the cycloaddition but is not observed here.^41^ A longer aliphatic segment corresponds to a greater distance between the furan groups and thus less steric hindrance for the bulky bismaleimide to reach the furan groups.

Finally, the conversions ranged from 28 to 45%, which are relatively high values. This means that 14 to 22% of the furan groups have reacted, on the basis of the conversion, furan-to-maleimide molar ratio and degree of polymerization. According to the Flory–Stockmayer theory, this should theoretically be sufficient to create a network structure and a gel-like material at the end of the reactions;^43^ however, this phenomenon was not observed. Therefore, it is expected that mainly intramolecular Diels–Alder reactions took place, which could be supported by the diluted system and the limited solubility of the reactants in DMSO. Under these conditions, the limited solubility hindered the formation of expanded polymer chains, as in the case of a good solvent, and therefore reduced the possibility for the bismaleimides to connect to other chains to take part as a cross-link. Similar experiments using chloroform, a good solvent for both reactants, did result in gel-like materials but hindered proper peak assignment due to overlapping signals. This indirectly demonstrates the success of the cross-linking step in chloroform, as this solvent promotes interchain cross-linking, enhancing cross-linking efficiency, and can be easily removed, unlike DMSO. In conclusion, the solvent and the casting procedure have a direct effect on the mechanical properties of the fabricated polymer sheet.

### Solubility Tests

The cross-linked materials were subjected to a solubility test to evidence the network formation and to determine the swelling ratio and gel content, and the results are presented in Table 2. None of the materials dissolved in the chloroform after immersion of 5 days at room temperature. In addition, all samples remained as one piece, except the PFSuc_0.5 samples which disintegrated into a few smaller particles. This implies that the cross-link density of PFSuc_0.5 is lower than the other materials, and is in line with the ^1^H NMR and TGA results. The swelling ratios are all in the same order of magnitude, which implies similar cross-link densities, but the swelling ratio is also affected by different polymer solvent interactions. The gel content of PFSuc_0.5 is lower compared to the other cross-linked materials, which is attributed to a lower degree of cross-linking and fragmentation of the samples reduced the accuracy of the measurements. The gel content of PFAd_0.5, PFSub_0.5 and PFSeb_0.5 are very close to or even higher than 100%. This indicates a very low soluble fraction of these materials and that not all the chloroform could be removed from the cross-linked materials during drying in the vacuum oven (24 h, 30 °C). In summary, these solubility tests confirm that intermolecular Diels–Alder reactions occurred between the furan and maleimide groups, resulting in the formation of insoluble cross-linked materials.

**Table 2:** Swelling Ratio and Gel Content of the Crosslinked Polymers with a Furan to Maleimide Molar Ratio of 1.0:0.5

material	swelling ratio	STD.	gel content (%)	STD.
PFSuc_0.5^a^	1.9	0.2	69	7
PFAd_0.5	2.2	0.1	104	1
PFSub_0.5	2.6	0.1	101	4
PFSeb_0.5	2.4	0.3	98	2

a The swelling ratio was based on two sample instead of three due to fragmentation of the third sample.

### Thermal Analysis and Recyclability

**Figure 5:**
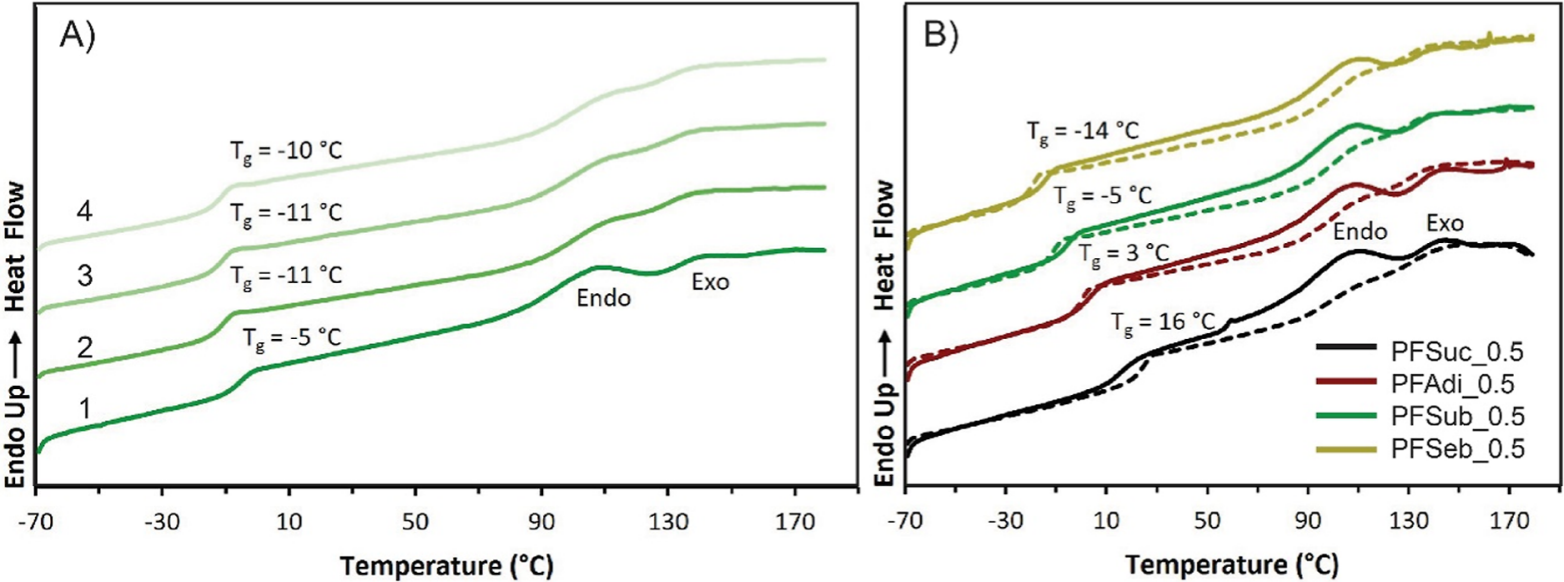
(A) DSC results of four heating curves of PFSub thermoreversibly cross-linked with BM-689 in a furan to maleimide molar ratio of 1.0:0.5, measured at a heating rate of 2 °C/min. (B) First two cycles of the four prepared films. The solid line represents the first heating curve, and the dashed line represents the second heating curve. The endothermic endo- and exotransitions are indicated.

The cross-linked materials of the BHMF-based polyesters were subjected to three heat–cooling cycles and one additional heating ramp. This was accomplished with a furan to maleimide molar ratio of 1.0:0.5. The DSC curves of the four cross-linked polymers are very similar; hence, the results of PFSub_0.5 are taken as an example and depicted in Figure 5A. The DSC results of PFSuc_0.5, PFAd_0.5 and PFSeb_0.5 are given in the Supporting Information (Figures S12–S14). As a result of Diels–Alder cross-linking, a shift in the *T*_g_ values can be observed between the linear polyesters and the prepared films. For PFSub, the *T*_g_ shifted from −28.5 to −5.3 °C for the cross-linked material as a consequence of the reduced segmental network mobility and flexibility caused by the cross-links. In addition, the increase in *T*_g_ value might also be attributed, in a minor extent, to intramolecular Diels–Alder reactions, which lead to large pendant groups and a reduced mobility.

The two different Diels–Alder endothermic transitions are clearly observed during the first heating curve, the onset of which occurs at 90 °C, with partial adduct cleavage of the endoadduct cleavage, followed by dissociation of exoadducts, initiating at approximately 130 °C.^28^ These peaks were still visible in the second, third and fourth heat curves but at lower magnitudes. This suggests a decreased amount of adduct cleavage in the second to fourth cycles and a change in the endo/exo-adduct ratio between the first and second steps, whereas this ratio remains identical in the third and fourth cycles. Hence, a dynamic equilibrium is expected to be established after these cycles, and the reversibility of the system remains unaffected after several heating cycles. The minor decrease in the *T*_g_ between the first and second cycles implies that cross-link conversion was lower in the second cycle because the retro-Diels–Alder occurred at the end of the first heating ramp. Consequently, the Diels–Alder conversion did not increase enough in the cooling step between cycles 1 and 2 to reach the preceding stage. Alternatively, the reduced *T*_g_ values could also be attributed to a relative increase in kinetically favorable endoadducts with respect to the exoadducts, which could influence the chain mobility and flexibility as well, although the impact is anticipated to be minimal.

Figure 5B illustrates the first and second heating curves of all four cross-linked materials and reveals that the thermal behavior is similar. The *T*_g_ value decreases with increasing length of the aliphatic segment, which is in line with the *T*_g_ values of the pure polyesters. The absence of melting points and the fact that retro-Diels–Alder initiates at approximately 90 °C indicate that PFAd_0.5, PFSub_0.5 and PFSeb_0.5 can be used at higher temperatures than the pure polymers because of the relatively low melting points of the pure polymers (Table 1).

Finally, the results imply that the cross-linked materials can be reprocessed via thermal treatment at least four times and that the Diels–Alder reaction in these systems is indeed reversible. The absence of an exothermic transition at temperatures above 130 °C reveals that side reactions such as maleimide homopolymerization,^44^ double Diels–Alder,^45^ or aromatization do not play a significant role here. This applies within the time frame during which the material is exposed to these temperatures.

**Figure 6:**
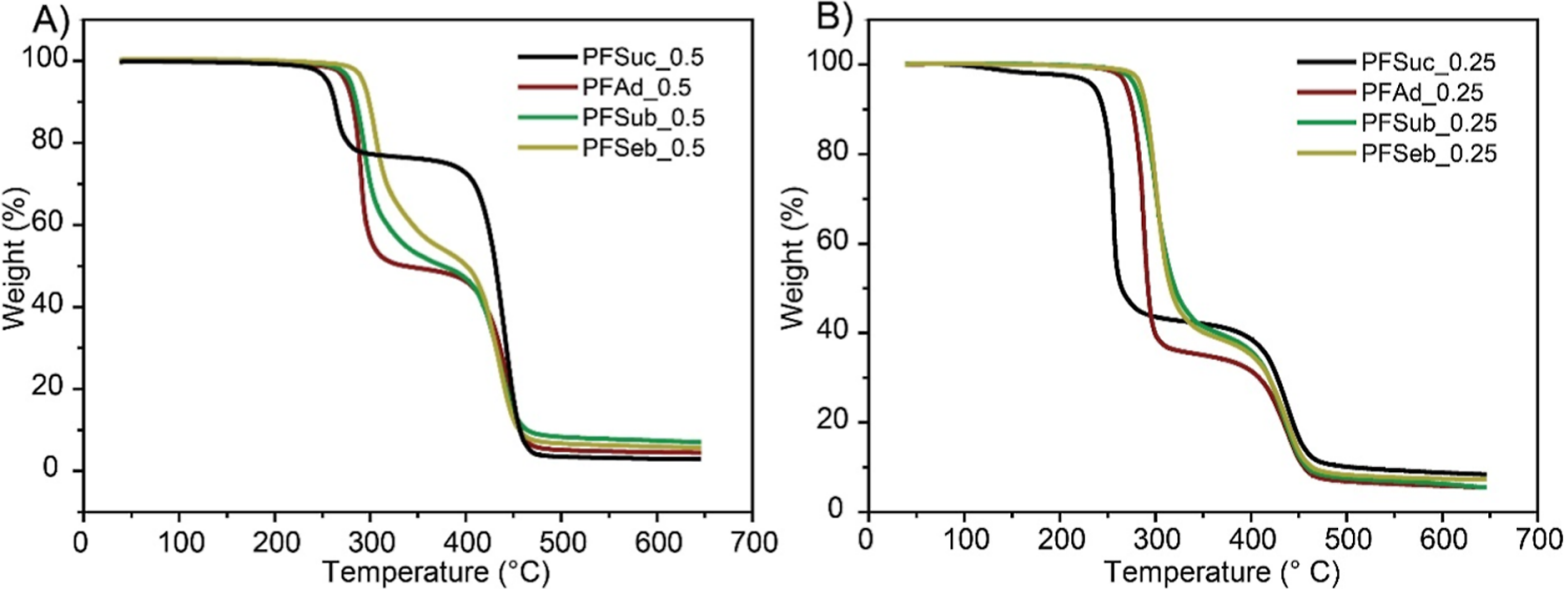
TGA results of cross-linked polymer–maleimide blends for (A) a furan/maleimide molar ratio of 1.0:0.5 and (B) a molar ratio of 1.0:0.25.

The thermal stability of the cross-linked materials was investigated via thermogravimetric analysis (TGA). The weight loss is displayed as a function of temperature in Figure 6 for two series of different furan-maleimide ratios. The first step in weight loss is attributed to polymer backbone degradation and corresponds well with the TGA results of the pure polymers (Table 1 and Figure S15). The second degradation step is caused by the degradation of bismaleimide (Figure S15). This indicates the absence of preceding cross-linked segments and implies that the materials are converted back to adducts before thermal degradation. Alternative side reactions, which can take place during the thermal heating step, such as homopolymerization,^44^ adduct aromatization reactions, or double Diels–Alder,^45^ did not alter the degradation temperature of the polymer chains and bismaleimide or led to a new degradation step. Notably, the PFSuc-based networks exhibit a slight inconsistency in the homogeneity of the samples, which is observed in the TGA curve because of the deviating weight loss of the first degradation step. This phenomenon is likely caused by the slower reaction kinetics of PFSuc, as shown in Figure 4, and by the partial crystallization of PFSuc during the cross-linking process as the solvent evaporates. This could be avoided by processing via reactive extrusion instead of solvent casting.

### Rheology

**Figure 7:**
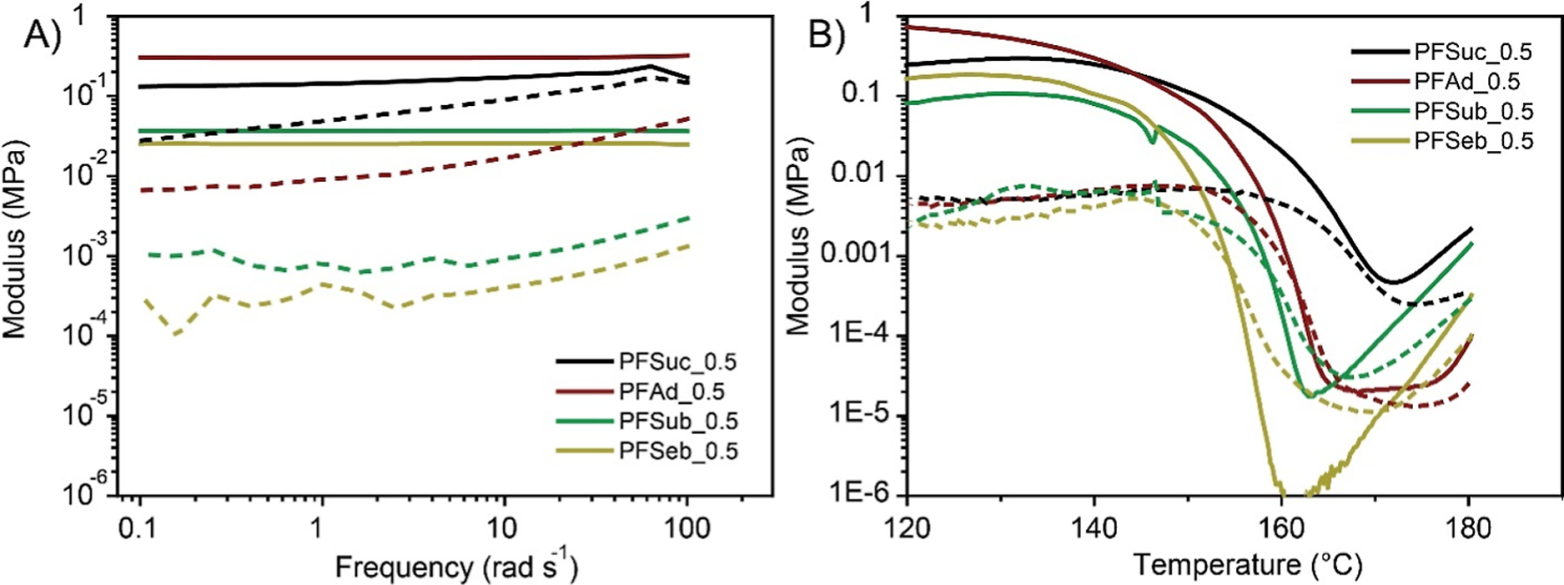
(A) Storage modulus and loss modulus of four prepared cross-linked polymers with a furan to maleimide molar ratio of 1.0:0.5 versus frequency, and (B) temperature upon a heating curve of 2 °C min^–1^ and constant frequency (1 Hz). The storage modulus is displayed as a solid line, and the loss modulus is displayed as a dashed line.

The mechanical behavior and recyclability of the dynamically cross-linked films were investigated via oscillatory rheometry. The cross-linked materials were subjected to a frequency sweep in the linear viscoelastic regime (Figure 7A) on the basis of the preceding strain sweep. All four samples behaved like solid-like viscoelastic materials since the storage moduli were higher than the loss moduli. In addition, the storage moduli remained rather independent of the frequency, which indicates a cross-linked structure. In contrast, the pure polyesters have a shear thinning behavior in the melt phase and a decreasing low frequency complex viscosity for an increasing spacer length.^11^ These results indicate that the mechanical properties are tunable, likely influenced by molecular weight, degree of cross-linking and choice of polyester.

With a dissociative mechanism, the retro-Diels–Alder destroys network integrity, stimulating macroscopic flow and reprocessability. In oscillatory rheometry, this can be observed in the form of a solid–liquid transition, with the crossover between the storage modulus and loss modulus being the gel point. This point is frequency independent according to the Winter–Chambon theorem.^46^ The results are displayed in Figure 7B. As evidenced by the crossover of the storage and loss moduli, the prepared polymer films of PFAd_0.5, PFSub_0.5, and PFSeb_0.5 revert to viscous liquids, with the degelation temperature decreasing with increasing furan spacer length. A similar phenomenon was observed for the pure polyesters, in which an increase in aliphatic segment size resulted in decreased complex viscosity.^11^ Notably, the magnitude of the storage moduli decreased by several orders of magnitude, which was attributed to the loss of network integrity.

However, at temperatures higher than 160 °C all the polymers seem to be prone to a new stiffening process. This phenomenon was previously observed for another bismaleimide and is attributed to maleimide homopolymerization, initiating at temperatures around 150 °C.^44,47^ Alternatively, aromatization of the adduct groups is discussed in literature, though not yet proven chemically.^48^ Lastly, double Diels–Alder (i.e., an additional cycloaddition between a furan and existing adduct) may be the reason for this behavior.^45^ One of these side reactions seems to be why PFSuc_0.5 did not undergo a solid–liquid transition. This material showed a decrease in both the storage and loss moduli but did not reach a crossover point. It is expected that this reinforcement process at higher temperatures started before the solid–liquid transition point could be reached. The solidification does not seem to be an exothermic process according to the DSC results but rather another process that jeopardizes the reversibility of the system.

These results imply the thermal and mechanical enhancement of these materials with respect to the pristine linear BHMF-based polyesters, which behave like viscous liquids above their limited melting points.^11^ All the cross-linked materials still behave like solid viscous-elastic materials up to a temperature of 150 °C, which is significantly higher than the melting points of the individual linear polymers. Therefore, these reversibly cross-linked materials are applicable in a broader thermal window of application and have improved mechanical performance.

## Conclusion

To overcome the limited thermal and mechanical properties of linear BHMF-based polyesters, the possibility of cross-linking the furan units in the polymer chains with a biobased aliphatic bismaleimide through [4 + 2] cycloaddition between furan and maleimide was investigated. Despite the possible steric hindrance of BHMF by the polymer chain itself, this work demonstrates that thermoreversible cross-linking in these systems can be realized, as proven by ^1^H NMR and IR spectroscopy. The rate at which this occurs appears to be dependent on the aliphatic spacer in the repeating unit. As evidenced by in situ ^1^H NMR studies in DMSO, a shorter aliphatic spacer hinders the Diels–Alder reaction to an extent, although high conversions are still achieved. In addition, retro-Diels–Alder was observed via differential scanning calorimetry, where the onset of the endothermic transition associated with this phenomenon occurred at temperatures similar to those of other furan–maleimide systems (>90 °C). DSC analysis revealed that the Diels–Alder network was thermoreversible and remained unaffected for at least 4 cycles. Thermal degradation of the material is observed at temperatures similar to those of the pure polymers and bismaleimide, which are well above 250 °C. Finally, the oscillatory rheology illustrates the mechanical properties and reprocessability of these materials, as the polymer network reverts from a solid-like material to a viscous liquid again upon heating. A longer aliphatic segment of the repeating unit leads, in this case, to a lower gelation temperature and thus facile recyclability compared to a shorter aliphatic spacer. All the polymer networks remained in a solid viscoelastic regime up to 150 °C, which is a significant increase compared with the limited melting point of the pure linear polymers. Solubility tests in chloroform evidenced the network formation of the prepared materials.

These results imply that BHMF-based polyesters can be thermally and mechanically enhanced by Diels–Alder cross-linking with BM-689. In addition, recyclability is maintained because of the thermoreversibility of the network. This widens the application window of these biobased and potentially biodegradable polyesters in the circular polymer economy.

## Supporting Information Available

Chemical structures of the polyesters and bismaleimide, COSY and HSQC spectra of the reaction products of PFSeb and BM-689, ^1^H NMR spectra of linear polyesters and reaction products of polymers and BM-689 after 16 h, DSC curves of cross-linked materials and TGA results of pure polyesters and BM-689 (PDF)
