# Supplementary material for: Thermoreversible Diels–Alder Cross-Linking of BHMF-Based Polyesters: Synthesis, Characterization and Rheology
Source: ACS Sustain Chem Eng. 2025 Feb 27;13(9):3543–53. doi: 10.1021/acssuschemeng.4c09338 (PMC11898174; doi:10.1021/acssuschemeng.4c09338)
Supplement: Supplementary file 1 — sc4c09338_si_001.pdf [file sc4c09338_si_001.pdf]

Supporting Information

Thermoreversible Diels-Alder crosslinking of BHMF-based polyesters: synthesis, characterization and rheology

Cornelis Post[‡1,2], Paul van den Tempel[‡3], Paula Herrera Sánchez[1,3], Dina Maniar[1], Ranjita K. Bose[3], Vincent S.D. Voet[2], Rudy Folkersma[2], Francesco Picchioni[3], Katja Loos[*1]
[‡]C. Post and P. van den Tempel contributed equally
[*]Corresponding author, Email. k.u.loos@rug.nl.

[1]Macromolecular Chemistry & New Polymeric Materials, University of Groningen, Nijenborgh 3, 9747 AG Groningen, the Netherlands
[2]Circular Plastics, NHL Stenden University of Applied Sciences, Van Schaikweg 94, 7811 KL Emmen, the Netherlands
[3]Department of Chemical Engineering, Engineering and Technology Institute Groningen (ENTEG), University of Groningen, Nijenborgh 3, 9747 AG Groningen, the Netherlands

Summary
This document contains the figures that illustrate the chemical structures of the polyesters and bismaleimide, COSY and HSQC spectra of the reaction products of PFSeb and BM-689, ^{1}H-NMR spectra of linear polyesters and reaction products of polymers and BM-689 after 16 h, DSC curves of crosslinked materials and TGA results of pure polyesters and BM-689.

Number of pages: 10

Number of figures: 15

Table of Contents

Figure S1. Chemical structures of the BHMF-based polyesters PFSuc, PFAd, PFSub and PFSeb and BM-689. ---------- S3

Figure S2. COSY spectrum of the reaction products of PFSeb and BM-689 in a furan to maleimide molar ratio of 1.0:0.5 after 16 h in DMSO-d_6 at 60 °C. ---------- S3

Figure S3. HSQC spectrum of the reaction products of PFSeb and BM-689 in a furan to maleimide molar ratio of 1.0:0.5 after 16 h in DMSO-d_6 at 60 °C. ---------- S4

Figure S4. ^{1}H-NMR spectrum of poly(2,5-furandimethylene succinate) (PFSuc) in DMSO-d_6. ---------- S4

Figure S5. ^{1}H-NMR spectrum of poly(2,5-furandimethylene adipate) (PFAd) in DMSO-d_6. ---------- S5

Figure S6. ^{1}H-NMR spectrum of poly(2,5-furandimethylene suberate) (PFSub) in DMSO-d_6. ---------- S5

Figure S7. ^{1}H-NMR spectrum of poly(2,5-furandimethylene sebacate) (PFSeb) in DMSO-d_6. ---------- S6

Figure S8. ^{1}H-NMR spectrum of the reaction products of PFSuc and BM-689 in a furan to maleimide molar ratio of 1.0:0.5 after 16 h in DMSO-d_6 at 60 °C. ---------- S6

Figure S9. ^{1}H-NMR spectrum of the reaction products of PFAd and BM-689 in a furan to maleimide molar ratio of 1.0:0.5 after 16 h in DMSO-d_6 at 60 °C. ---------- S7

Figure S10. ^{1}H-NMR spectrum of the reaction products of PFSub and BM-689 in a furan to maleimide molar ratio of 1.0:0.5 after 16 h in DMSO-d_6 at 60 °C. ---------- S7

Figure S11. ^{1}H-NMR spectrum of the reaction products of PFSeb and BM-689 in a furan to maleimide molar ratio of 1.0:0.5 after 16 h in DMSO-d_6 at 60 °C. ---------- S8

Figure S12. DSC curves of four heating cycles of PFSuc thermoreversibly crosslinked with BM-689 in a furan to maleimide molar ratio of 1.0:0.5 at a rate of 2 °C/min. ---------- S8

Figure S13. DSC curves of four heating cycles of PFAd thermoreversibly crosslinked with BM-689 in a furan to maleimide molar ratio of 1.0:0.5 at a rate of 2 °C/min. ---------- S9

Figure S14. DSC curves of four heating cycles of PFSeb thermoreversibly crosslinked with BM-689 in a furan to maleimide molar ratio of 1.0:0.5 at a rate of 2 °C/min. ---------- S9

Figure S15: Thermogravimetric analysis of the BHMF-based polyesters PFSuc, PFAd, PFSub, PFSeb and BM-689. ---------- S10

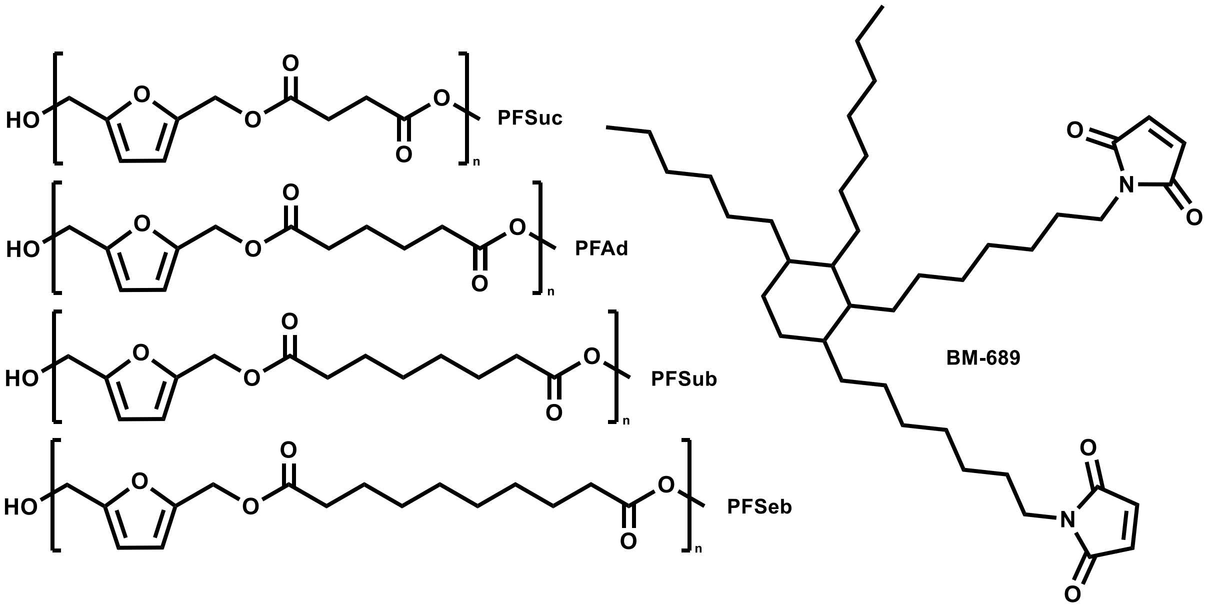

Figure S1. Chemical structures of the BHMF-based polyesters PFSuc, PFAd, PFSub and PFSeb and BM-689.

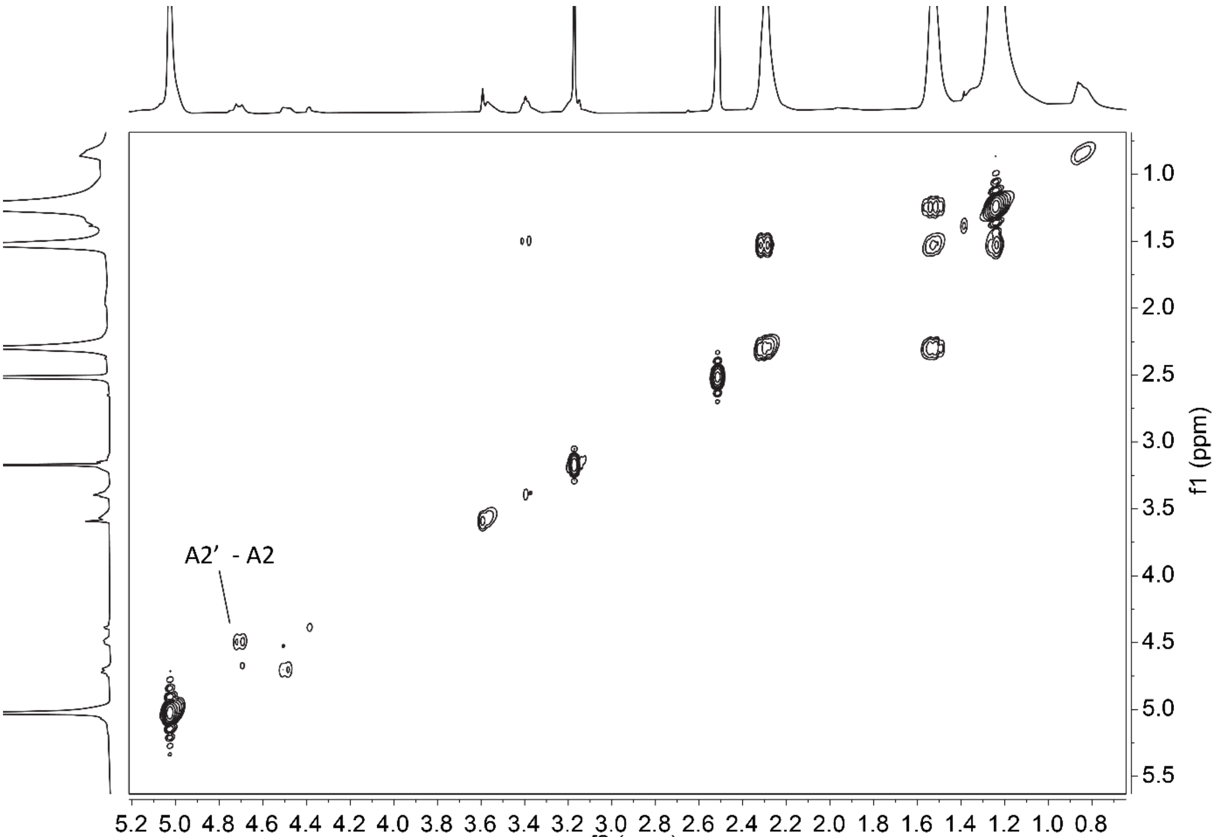

Figure S2. COSY spectrum of the reaction products of PFSeb and BM-689 in a furan to maleimide molar ratio of 1.0:0.5 after 16 h in DMSO-d_6 at 60 °C.

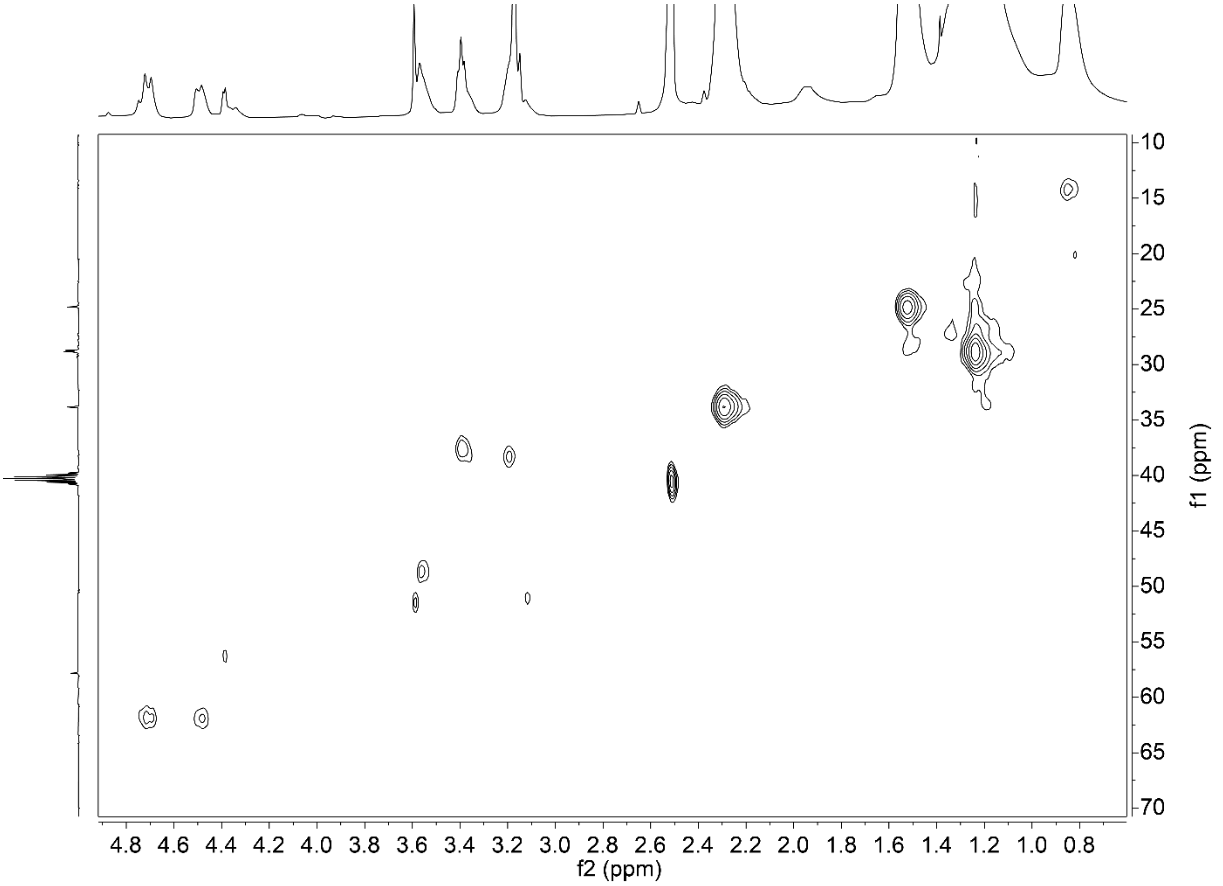

Figure S3. HSQC spectrum of the reaction products of PFSeb and BM-689 in a furan to maleimide molar ratio of 1.0:0.5 after 16 h in DMSO-d_6 at 60 °C.

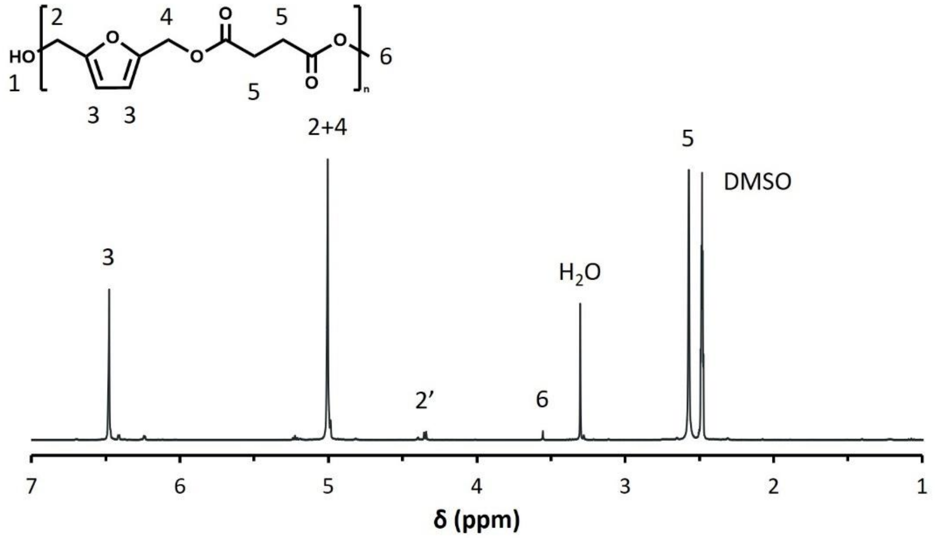

Figure S4. ^{1}H-NMR spectrum of poly(2,5-furandimethylene succinate) (PFSuc) in DMSO-d_6.

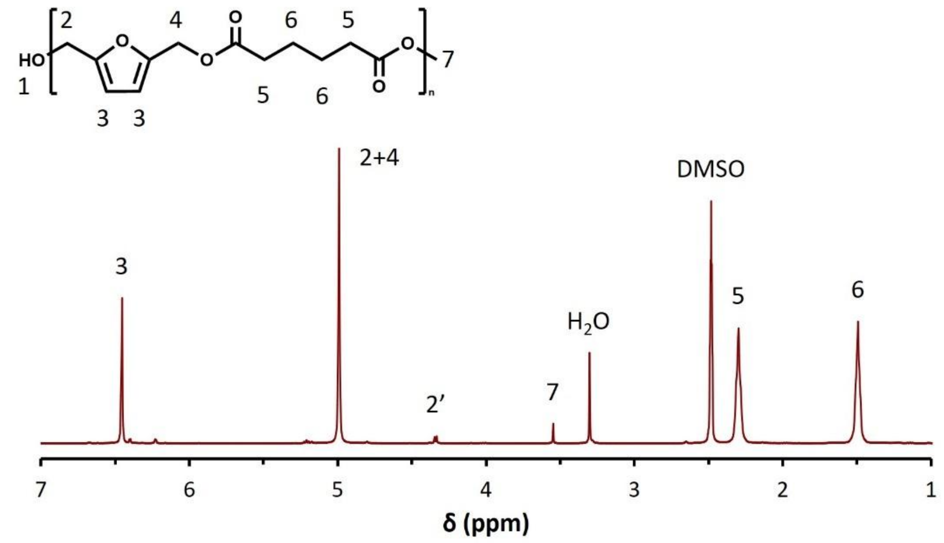

Figure S5. ^{1}H-NMR spectrum of poly(2,5-furandimethylene adipate) (PFAd) in DMSO-d_6.

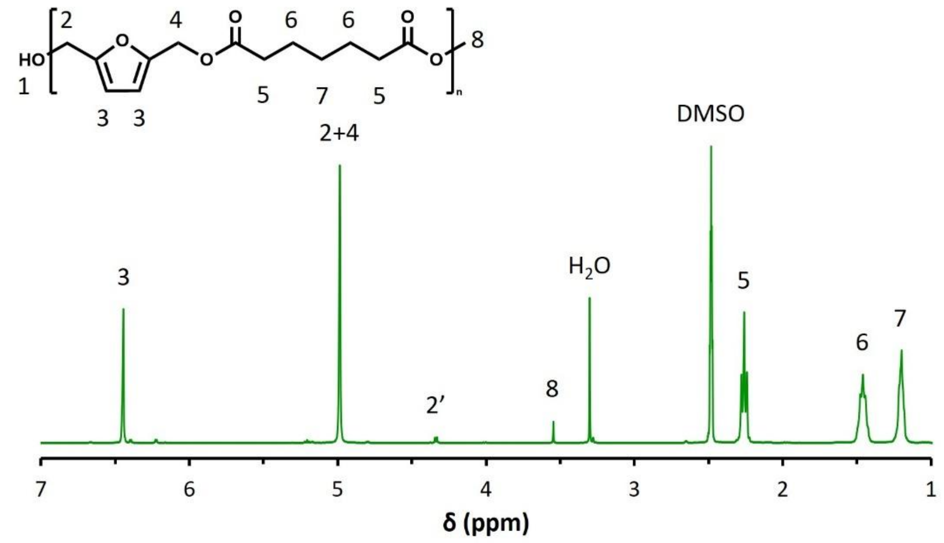

Figure S6. ^{1}H-NMR spectrum of poly(2,5-furandimethylene suberate) (PFSub) in DMSO-d_6.

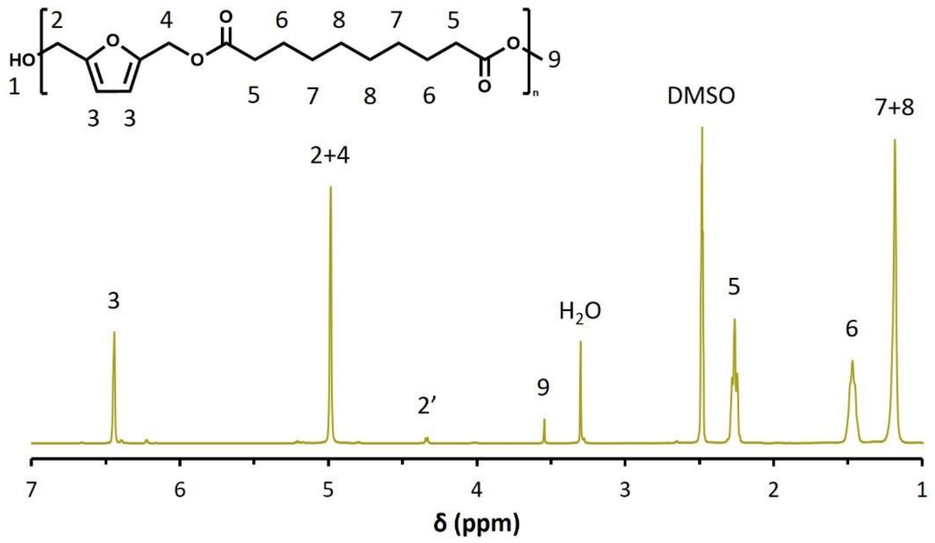

Figure S7. ^{1}H-NMR spectrum of poly(2,5-furandimethylene sebacate) (PFSeb) in DMSO-d$_6$.

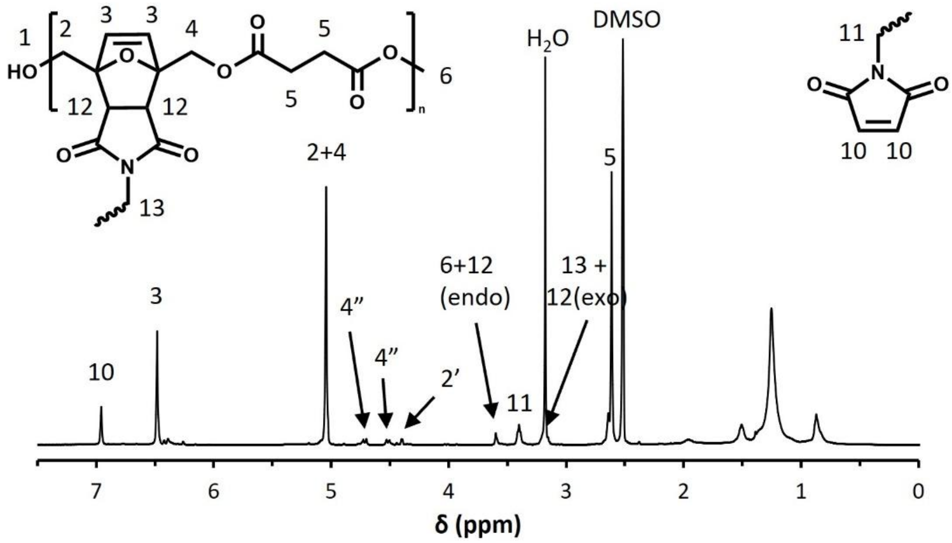

Figure S8. ^{1}H-NMR spectrum of the reaction products of PFSuc and BM-689 in a furan to maleimide molar ratio of 1.0:0.5 after 16 h in DMSO-d$_6$ at 60 °C.

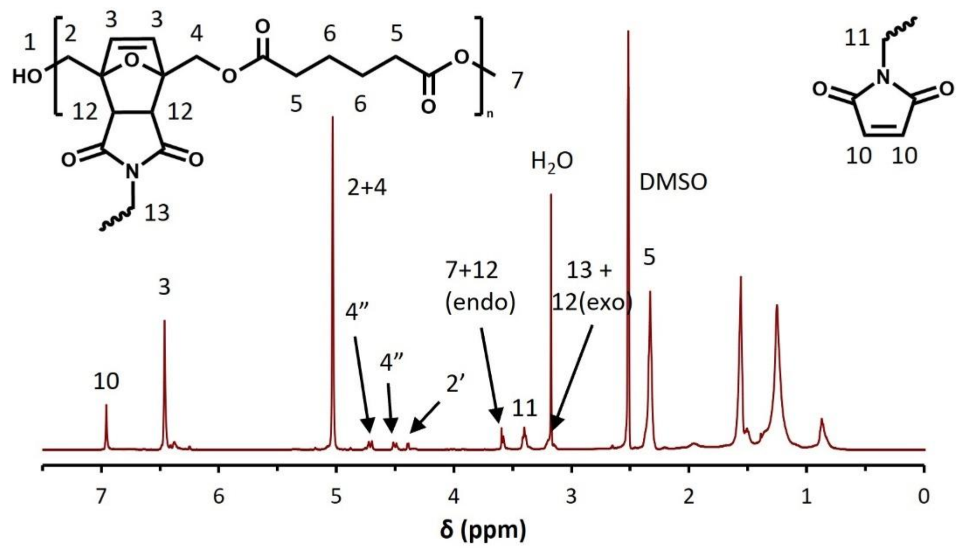

Figure S9. ^{1}H-NMR spectrum of the reaction products of PFAd and BM-689 in a furan to maleimide molar ratio of 1.0:0.5 after 16 h in DMSO-d_6 at 60 °C.

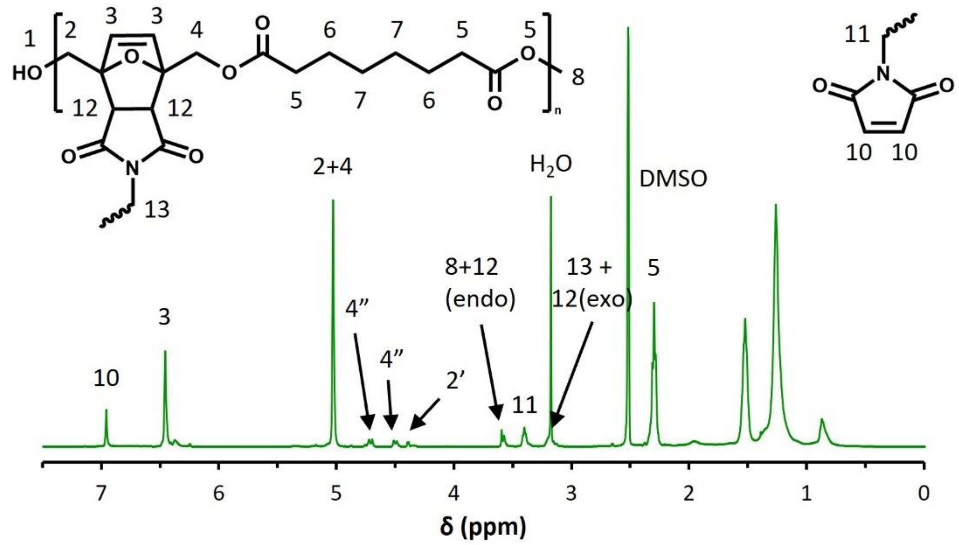

Figure S10. ^{1}H-NMR spectrum of the reaction products of PFSub and BM-689 in a furan to maleimide molar ratio of 1.0:0.5 after 16 h in DMSO-d_6 at 60 °C

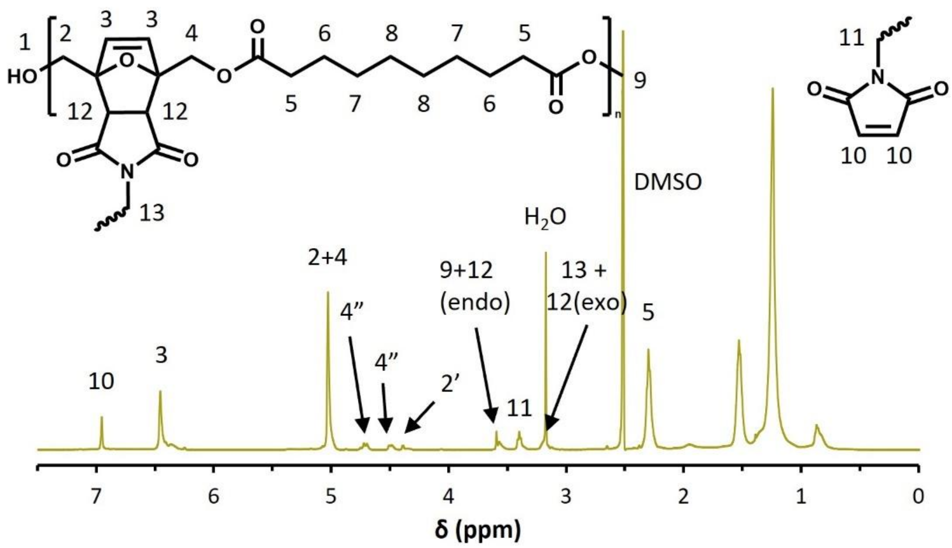

Figure S11. ^{1}H-NMR spectrum of the reaction products of PFSeb and BM-689 in a furan to maleimide molar ratio of 1.0:0.5 after 16 h in DMSO-d_6 at 60 °C

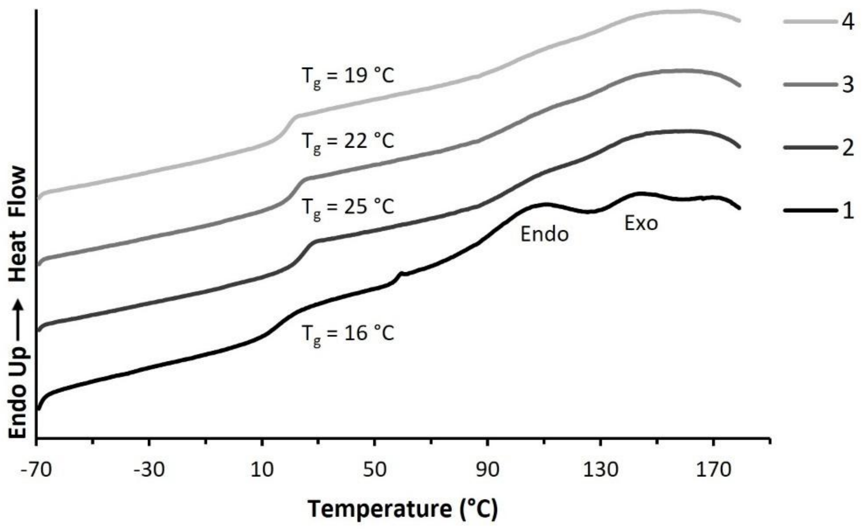

Figure S12. DSC curves of four heating cycles of PFSuc thermoreversibly crosslinked with BM-689 in a furan to maleimide molar ratio of 1.0:0.5 at a rate of 2 °C/min.

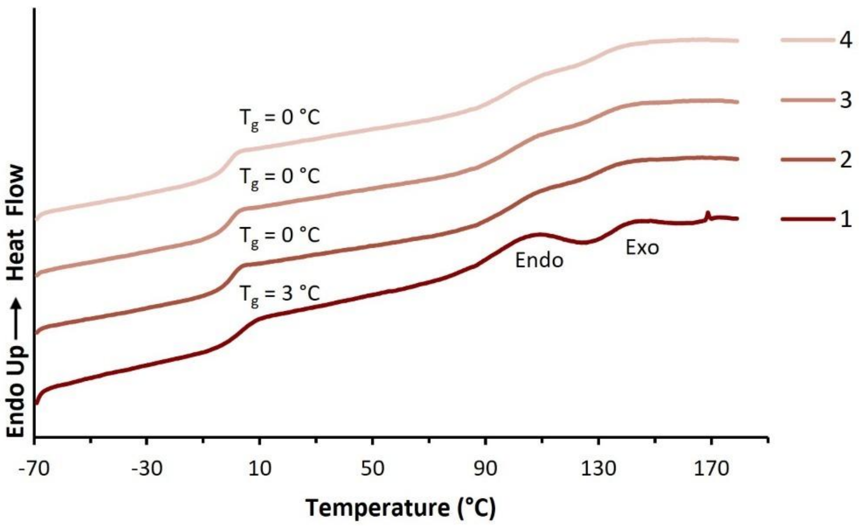

Figure S13. DSC curves of four heating cycles of PFAd thermoreversibly crosslinked with BM-689 in a furan to maleimide molar ratio of 1.0:0.5 at a rate of 2 °C/min.

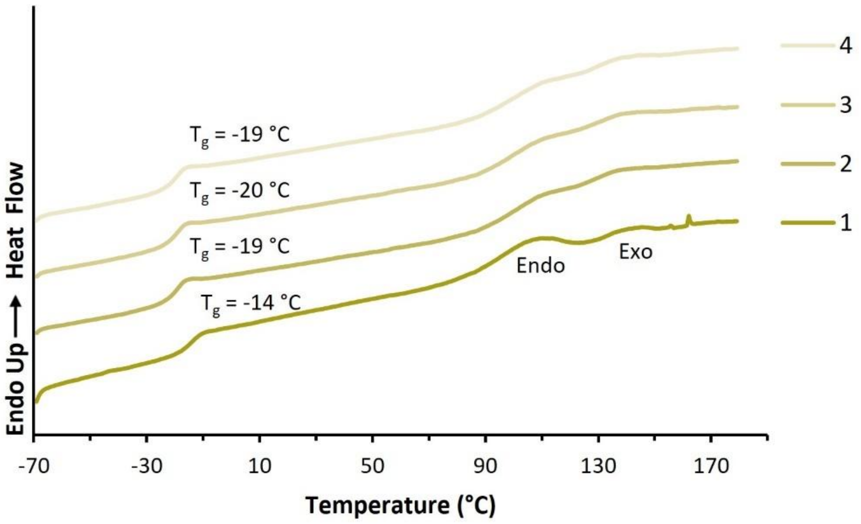

Figure S14. DSC curves of four heating cycles of PFSeb thermoreversibly crosslinked with BM-689 in a furan to maleimide molar ratio of 1.0:0.5 at a rate of 2 °C/min.

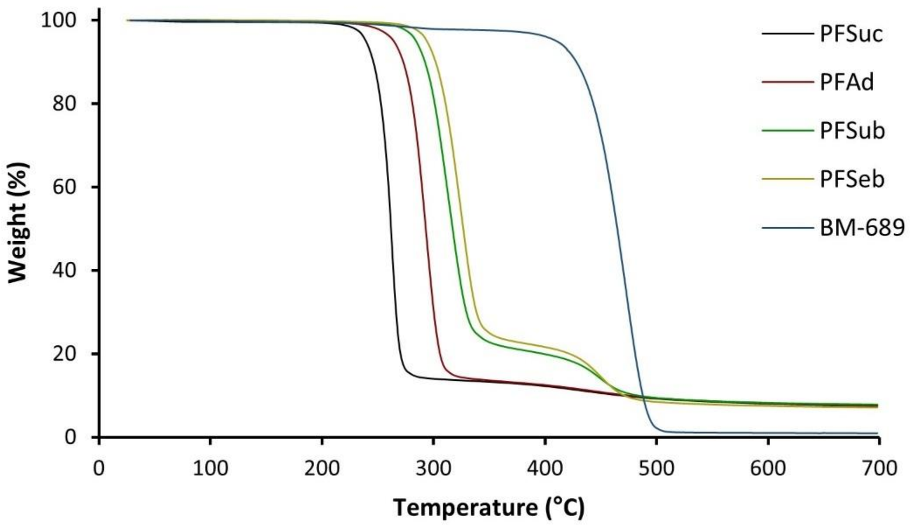

Figure S15: Thermogravimetric analysis of the BHMF-based polyesters PFSuc, PFAd, PFSub, PFSeb and BM-689.
